# Melioidosis Acquired by Traveler to Nigeria

**DOI:** 10.3201/eid1707.110502

**Published:** 2011-07

**Authors:** Alex P. Salam, Nisa Khan, Henry Malnick, Dervla T.D. Kenna, David A.B. Dance, John L. Klein

**Affiliations:** Author affiliations: Guy’s and St. Thomas’ National Health Service Trust, London, UK (A.P. Salam, N. Khan, J.L. Klein);; Health Protection Agency, London, UK (H. Malnick, D.T.D. Kenna, D.A.B. Dance);; Mahosot Hospital, Vientiane, Laos (D.A.B. Dance);; University of Oxford, Oxford, UK (D.A.B. Dance);; Churchill Hospital, Oxford (D.A.B. Dance)

**Keywords:** Burkholderia pseudomallei, melioidosis, Africa, Nigeria, eBURST, diabetes, bacteria, dispatch

## Abstract

We describe melioidosis associated with travel to Nigeria in a woman with diabetes, a major predisposing factor for this infection. With the prevalence of diabetes projected to increase dramatically in many developing countries, the global reach of melioidosis may expand.

## The Patient

A 46-year-old woman sought treatment in the emergency department of St Thomas’ Hospital, London, UK. She described a 2-day history of frontal headache, fever, and painful swelling in the right postauricular region. She had received a diagnosis of type 2 diabetes mellitus 5 months before admission, for which she took metformin (500 mg 2×/d). She did not smoke or drink alcohol. She was born in Ogun State, Nigeria, and had moved to the United Kingdom at the age of 31 years. She recently had visited relatives in Sagamu City, Ogun State, and returned to the United Kingdom 6 weeks before onset of her illness. She had no history of travel outside the United Kingdom or Nigeria.

Examination at admission indicated a temperature of 39.5°C, a heart rate of 108 beats per minute, and a blood pressure level of 132/84 mm Hg. Other examination findings were unremarkable, apart from a tender, firm, erythematous, and hot swollen area (4 cm × 3 cm) in the right postauricular region. Initial investigations found a leukocyte count of 6.1 × 10^9^ cells/L (neutrophils 4.4 × 10^9^ cells/L) and a C-reactive protein level of 406 mg/L; renal and liver profiles were normal. Results of a hemoglobin A1c blood test for diabetes were 13%, which is consistent with poorly controlled diabetes, and results of an HIV test and malaria screen were both negative. Ultrasonography of the swollen area showed localized, superficial, enlarged lymph nodes, posterior and inferior to the right pinna. Blood was drawn for culturing, and a course of intravenous flucloxacillin treatment was begun. Subsequently, the aerobic bottles of 2 sets of blood cultures were positive for gram-negative bacilli, and subculturing yielded an oxidase-positive, gram-negative bacillus that grew rapidly on blood agar as a gray colony with a metallic sheen. The organism was subsequently identified as *Burkholderia pseudomallei* (see characterization of blood culture isolate). The antimicrobial drug therapy was changed to intravenous co-amoxiclav (1.2 g 3×/d), and 3 days later, when the identification of the organism was confirmed, the patient’s treatment was switched to intravenous meropenem (2 g 3×/d). Results of a computed tomography scan of the chest and abdomen were normal. The patient’s fever and lymphadenopathy subsequently resolved, and after 9 days of receiving meropenem, she was discharged with a 12-week course of oral co-trimoxazole (1,920 mg daily). The patient did not attend her scheduled outpatient appointment.

Two microbiology laboratory workers were judged to have had low-risk exposure to the organism before its identification (*1*). They were counseled, and both chose not to receive antimicrobial drug prophylaxis. Serologic follow up at 2 and 6 weeks’ postexposure for 1 worker showed no evidence of seroconversion to *B. pseudomallei*. The other did not attend her scheduled occupational health outpatient appointment.

Because the isolate could not be definitively identified by using API20NE (bioMérieux, Marcy l’Etoile, France), it was sent to the UK Health Protection Agency reference laboratory for further analysis. The appearance of the organism in colonies on Ashdown agar was typical of *B. pseudomallei*. Moreover, the same identification was reached through fatty acid methyl ester analysis by gas chromatography with the rapid bioterrorism database (MIDI Sherlock, Newark, NJ, USA). PCR amplification, using primers that specifically detect the type III secretion system gene cluster of *B. pseudomallei* (*2*), produced the predicted 115-bp band for our isolate and the positive control, *B. pseudomallei* strain 204 (*3*). Multilocus sequence typing, performed by using PCR amplification conditions and primers as previously described (*3*), generated an allelic profile (1, 1, 10, 2, 5, 2, 1) that was compared with those of strains of *B. pseudomallei*, *B. mallei*, and *B. thailandensis* stored in the database (http://bpseudomallei.mlst.net). This profile was unique among strains in the multilocus sequence typing database and therefore represents a novel sequence type (ST), ST707. An evolutionary analysis of ST707 with eBURST version 3 (http://eburst.mlst.net) suggested that it was most closely related to other strains of *B. pseudomallei,* including ST26 from Niger, ST82 from France, and ST7 from Vietnam (Figure).

**Figure:**
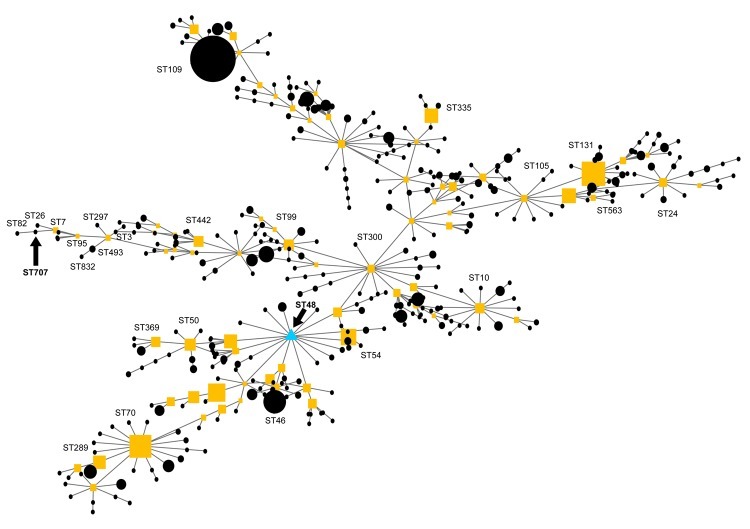
eBURST analysis showing the evolutionary relationship of sequence type (ST) 707 to *Burkholderia pseudomallei*, *B. mallei*, and *B. thailandensis* strains on the multilocus sequence typing database. Each circle and square (the latter indicate subfounders) represents an ST, with size being proportional to the number of isolations of that particular ST. The primary founder is ST48 (triangle). The lines directly linking STs represent strains that differ at a single locus in their allelic profile.

*B. pseudomallei* is the causative agent of melioidosis, a severe illness usually characterized by fever, lymphadenopathy, and suppurative lesions in skin, liver, spleen or the lung. The organism is present in the soil in disease-endemic areas, and infection is thought to be acquired through breaks in the skin. The disease is endemic to many parts of Southeast Asia, with most cases being reported from Thailand (*4*). The infection is also a well-recognized pathogen in the Northern Territory of Australia (*5*). In addition, sporadic cases have been described in Central and South America and the Pacific islands. By contrast, strikingly few human cases of melioidosis have previously been reported in Africa. Wall et al. reported a case in The Gambia in a patient originally from Sierra Leone (*6*), and Bremmelgaard et al. noted a case in a Danish patient who had probably acquired the infection in Kenya (*7*). Despite the paucity of clinical cases reported from Africa, serologic surveys suggest that the organism is present in several countries, including Burkina Faso (*8*) and Uganda (*9*). Moreover, *B. pseudomallei* has been isolated from the soil and from animals in several African countries (*10**,**11*).

## Conclusions

Although we cannot be certain, the patient reported here likely acquired her infection in Nigeria, the most populous country on the African continent. To our knowledge, no documented cases of melioidosis acquired in Nigeria have been reported. Most cases imported into the United Kingdom originate in Southeast Asia and the Indian subcontinent (*12*). Given the frequency of travel between the United Kingdom and Nigeria, the absence of imported cases from that country until now is striking. However, the patient’s strain appears to be related to a strain that was isolated from neighboring Niger ≈40 years earlier. Although the case we report here may represent a recent introduction of the organism into Nigeria, the disease may also be underdiagnosed because of the difficulty of identifying the organism in a resource-limited setting.

Although melioidosis may affect healthy persons, infection is strongly associated with underlying diseases and with diabetes in particular. In a case–control study in Thailand, diabetes was associated with an adjusted odds ratio of 12.9 (*13*). With the prevalence of diabetes projected to increase dramatically over the next few decades in the developing world (*14*), melioidosis may become more common in countries such as Nigeria. Finally, from a clinical standpoint, the patient reported here highlights the need to consider the diagnosis of melioidosis, even in the absence of travel to traditional disease-endemic countries.

## References

[1] Peacock SJ, Schweizer HP, Dance DA, Smith TL, Gee JE, Wuthiekanun V, Management of accidental laboratory exposure to *Burkholderia pseudomallei* and *B. mallei.* Emerg Infect Dis. 2008;14:e2. 10.3201/eid1407.071501 18598617 PMC2600349

[2] Novak RT, Glass MB, Gee JE, Gal D, Mayo MJ, Currie BJ, Development and evaluation of a real-time PCR assay targeting the type III secretion system of *Burkholderia pseudomallei.* J Clin Microbiol. 2006;44:85–90. 10.1128/JCM.44.1.85-90.2006 16390953 PMC1351940

[3] Godoy D, Randle G, Simpson AJ, Aanensen DM, Pitt TL, Kinoshita R, Multilocus sequence typing and evolutionary relationships among the causative agents of melioidosis and glanders, *Burkholderia pseudomallei* and *Burkholderia mallei.* J Clin Microbiol. 2003;41:2068–79. 10.1128/JCM.41.5.2068-2079.2003 12734250 PMC154742

[4] Vuddhakul V, Tharavichitkul P, Na-Ngam N, Jitsurong S, Kunthawa B, Noimay P, Epidemiology of *Burkholderia pseudomallei* in Thailand. Am J Trop Med Hyg. 1999;60:458–61. 10466977 10.4269/ajtmh.1999.60.458

[5] Currie BJ, Fisher DA, Howard DM, Burrow JN, Lo D, Selva-Nayagam S, Endemic melioidosis in tropical northern Australia: a 10-year prospective study and review of the literature. Clin Infect Dis. 2000;31:981–6. 10.1086/318116 11049780

[6] Wall RA, Mabey DC, Corrah PT, Peters L. A case of melioidosis in West Africa. J Infect Dis. 1985;152:424–5. 10.1093/infdis/152.2.424a 4031552

[7] Bremmelgaard A, Bygbjerg I, Hoiby N. Microbiological and immunological studies in a case of human melioidosis diagnosed in Denmark. Scand J Infect Dis. 1982;14:271–5. 7163779 10.3109/inf.1982.14.issue-4.05

[8] Dodin A, Ferry D, Sanson R, Guenole A. Découverte du bacille de Whitmore en Afrique: compte-rendu de mission. Med Mal Infect. 1975;5:97–101. 10.1016/S0399-077X(75)80036-9

[9] Frazer DN. Melioidosis. J R Army Med Corps. 1982;128:123–30. 7175789 10.1136/jramc-128-03-02

[10] Galimand M, Dodin A. Le point sur la melioidose dans le monde. [**PMID: 7172358**]. Bull Soc Pathol Exot. 1982;75:375–83. 7172358

[11] Ferry NR. Isolement du bacille de Whitmore a partir de lesions rencontrees chez le porc a l’abattoir de Niamey au Niger. Bull Soc Pathol Exot. 1973;66:42–5. 4801790

[12] Malnick H, Englender HA, Dance DAB, Smith MD, Simpson AJH, Pitt TL. A decade of experience of United Kingdom’s melioidosis diagnostic service. 5th World Melioidosis Congress, Khon Kaen, Thailand, November 21–23, 2007 [cited 2011 May 24]. http://www.hpa.org.uk/web/HPAwebFile/HPAweb_C/1203496926848

[13] Suputtamongkol Y, Chaowagul W, Chetchotisakd P, Lertpatanasuwun N, Intaranongpai S, Ruchutrakool T, Risk factors for melioidosis and bacteremic melioidosis. Clin Infect Dis. 1999;29:408–13. 10.1086/520223 10476750

[14] Mbanya JC, Motala AA, Sobngwi E, Assah FK, Enoru ST. Diabetes in sub-Saharan Africa. Lancet. 2010;375:2254–66. 10.1016/S0140-6736(10)60550-8 20609971

